# Multisystem Inflammatory Syndrome in Children (MIS-C)—A Case Series in December 2020 in Vienna, Austria

**DOI:** 10.3389/fped.2021.656768

**Published:** 2021-06-10

**Authors:** Herbert Kurz, Tomas Gombala

**Affiliations:** Department of Pediatrics, Clinic Donaustadt, Vienna, Austria

**Keywords:** multisystem inflammatory syndrome in children, pediatric inflammatory multisystem syndrome temporally associated with SARS-CoV-2/COVID-19 infection, COVID-19, SARS-CoV-2, Kawasaki-like

## Abstract

MIS-C is a novel clinical syndrome in children and adolescents, was first encountered in the spring of 2020 as a post COVID-19 multisystem inflammatory syndrome. The highest number of SARS-CoV-2 infections in Austria were registered in November 2020. In December 2020, eight patients with MIS-C were hospitalized at the pediatric department of Klinik Donaustadt, a large municipal hospital in Vienna. Six of the patients needed pediatric intensive care. As we lack any international consensus on the diagnosis and treatment of this severe complication, we used a multidisciplinary approach. The purpose was to establish an internal standard for maximizing the efficacy and safety of treatment, and standardizing the clinical management of this disease.

## Introduction

MIS-C is a novel clinical syndrome that first appeared in 2020 (1–4). A mild or even asymptomatic acute SARS-CoV-2 infection in children and adolescents is followed ~2–4 weeks later by a life-threatening condition. This new syndrome of hyperinflammation currently lacks a specific name. Alternatives include pediatric hyperinflammatory syndrome, pediatric hyperinflammatory shock, pediatric multisystem inflammatory syndrome (PMIS), multisystem inflammatory syndrome in children (MIS-C), or pediatric inflammatory multisystem syndrome temporally associated with SARS-CoV-2 (PIMS-TS). The pathogenesis is not fully elucidated yet. Research suggests a postinfectious immune dysregulation, such as uncontrolled T-cell mediated immune response with a cytokine storm and the production of multiple autoantibodies. Potential triggers could be superantigens binding to T-cell receptors (αβTCRs) in combination with a genetic predisposition, such as specific HLA types. Some studies found lower naive T-helper cells, and increases in central and effector memory subpopulations. Furthermore, dysregulation of T cells is suggested through expanded CD57+ CD4+ T cells. IL17A-mediated inflammation was seen more in Kawasaki disease than in MIS-C (1, 5, 6). The syndrome partly mimics Kawasaki disease, toxic shock syndrome, and hemophagocytic lymphohistiocytosis (HLH). The syndrome partly mimics Kawasaki disease, toxic shock syndrome, and hemophagocytic lymphohistiocytosis (HLH). Based on current knowledge, two descriptive definitions have been published. One of these is MIS-C {established by the WHO (7) and the US Center for Disease Control and Prevention (CDC) (8)}, which includes the following criteria: fever for more than 3 days, age under 20 years, two or more signs of multisystemic involvement (such as hypotension, GIT symptoms, rash, conjunctivitis, coagulopathy, etc.), elevated markers of inflammation, evidence of COVID-19 infection or exposure, and exclusion of other causes of inflammation. The second definition is known as PIMS-TS and was established in the UK by the Royal College of Paediatrics and Child Health (RCPCH) (9). It divides the syndrome into two phenotypes: Kawasaki-disease-like and non-specific (10). We adopted the WHO definition of MIS-C, which includes symptoms of both phenotypes. All patients were diagnosed and treated in accordance with a local standard, taking the numerous aspects of the disease into account.

## Cases

In December 2020, eight patients ranging in age from 2 years and 4 months to 18 years and 7 months (median 11 years and 2 months) were admitted to the pediatric ward of the hospital (Table 1). Three patients were female and five were male. Six of them were hospitalized simultaneously. Three were transferred from another hospital. One patient was African, one of Indian origin, two were of Arabian descent, and the others from Central Europe. Seven patients had no underlying comorbidities, one patient had undergone heart surgery 8 and 9 years ago (pulmonary banding and resection of a bronchogenic cyst as a newborn and VSD closure 1 year later). All patients had tested negative on the SARS-CoV-2 rapid-antigen test, and also tested negative on the RT-PCR test of their nasopharyngeal swabs at the time of admission.

**Table 1:** Patient characteristics.

**Characteristics**	**Case 1**	**Case 2**	**Case 3**	**Case 4**	**Case 5**	**Case 6**	**Case 7**	**Case 8**
Sex	Female	Male	Male	Male	Male	Female	Male	Female
Age	15 6/12	2 4/12	12 0/12	8 3/12	14 7/12	10 5/12	18 7/12	9 2/12
Ethnicity	Central European	Central European	Central European	Arabian origin	Indian origin	Arabian origin	Central European	African
Days in the PICU	7	0	7	0	2	28	4	9
Days in the hospital	11	11	15	16	10	54	9	15
**Presenting symptoms**								
No. of days with fever before admission	6	1	5	3	4	0	6	5
Abdominal pain	**Yes**	No	**Yes**	**Yes**	**Yes**	**Yes**	No	**Yes**
Signs of appendicitis	**Yes**	No	**Yes**	**Yes**	No	No	No	No
Emesis	**Yes**	No	**Yes**	**Yes**	No	No	No	**Yes**
Diarrhea	**Yes**	No	**Yes**	No	**Yes**	No	No	**Yes**
Testicular pain		No	No	No	**Yes**		No	
Febrile seizure	No	**Yes**	No	No	No	No	No	No
Dry cough	No	**Yes**	**Yes**	No	No	No	No	No
Sore throat	No	No	No	No	No	No	**Yes**	**Yes**
Headache	No	No	No	No	No	No	No	**Yes**
**History of acute SARS-CoV-2 infection**								
History of acute SARS-CoV-2 infection (including positive test result)	No	No	No	Yes−4 weeks prior to admission, fever, sore throat	No	No	Yes−3 weeks prior to admission, asymptomatic	Yes−2 weeks prior to admission
History of acute SARS-CoV-2 infection in the family	Brother tested positive 1.5 months prior to admission	No	Parents tested positive 2–3 weeks prior to admission	No	Father and sister tested positive 1.5 months prior to admission	No	No	No
**Laboratory values before the start of treatment**								
WBC (4.8–12.0 G./l)	13.8	7	14.5	9.2	7.9	12.5	9.5	15.9
Neutrophils (rel. 33–74%)	88.8	76.2	92	91.4	85	87.5	85.5	95.6
Lymphocytes (rel. 22–51%)	9	14.4	4	4.6	4	5.4	6.9	1.7
Platelets (180–415 G./l)	70	116	92	80	20	146	128	155
Ferritin (7–140 μg/l)	447	413	827	665	378	162	1,250	611
ESR	108/129	30/53	Not done	40/65	65/90	Not done	66/91	>119 in 1 h
CRP (mg/l)	200	90	478.8	249.6	229.7	215	205.6	398.4
IL-6 (pg/ml)	309.6	212.8	>5,000	291.8	1,591	1,198	18.8	1,546
Procalcitonin (ng/ml)	5.18	Not done	142.8	10.89	47.53	17.5	10.8	39.8
D-dimer (<0.5 mg/l)	2.76	5.55	15.38	4.4	5.91	2.67	4.6	4.4
Hs-Trop-I (<53.7 ng/l)	1,433.1	neg	480.6	34.7	1,124.5	20,308	258.2	264
Pro-BNP (<155 ng/l)	8,997	5,999	88,759	3,099	10,739	24,580	15,293	89,367
Albumin (41–48 g/l)	22	31	18	26	19	16	19	23
SARS-CoV-2 RT-PCR	Positive (CT 37), Negative	Negative	Negative	Negative	Negative	Negative	Negative	Negative
SARS-CoV-2 IgG antibody U/ml (>15 positive)	105	112	80.3	121	90.9	41.9	74.9	61.2
**PICU**	**Yes**	No	**Yes**	No	**Yes**	**Yes**	**Yes**	**Yes**
Catecholamines	**Yes**		**Yes**		**Yes**	**Yes**	**Yes**	**Yes**
Nasal high-flow	**Yes**		**Yes**		No	No	**Yes**	**Yes**
Invasive ventilation	No		No		No	**Yes**	No	No
**Outcome 3 weeks after discharge**								
Feels well	**Yes**	**Yes**	**Yes**	**Yes**	**Yes**	**Yes**	**Yes**	**Yes**
Any signs of sequelae	No	No	No	No	No	No	No	No

The patients' symptoms at admission are listed in Table 2. Six patients had to be transferred or were admitted directly to the pediatric intensive care unit, in most cases because they needed catecholamines due to hypotension and/or signs of shock. Five patients needed respiratory support; one of them received invasive mechanical ventilation, four needed nasal high-flow therapy, and one required nasal oxygen. The median duration of stay at the pediatric ICU was 7 days (range, 2–28 days). All patients fulfilled the MIS-C criteria defined by the WHO.

**Table 2:** Clinical symptoms at admission.

Fever; duration of fever	7/8 (87.5%); 1–6 days, median 4.5 days
Abdominal pain	6/8 (75%)
Emesis	4/8 (50%)
Diarrhea	4/8 (50%)
Signs of appendicitis	3/8 (37.5%)
Dry cough	2/8 (25%)
Sore throat	2/8 (25%)
Testicular pain	1/5 (20%)
Febrile seizure	1/8 (12.5%)
Headache	1/8 (12.5%)

**Figure 1:**
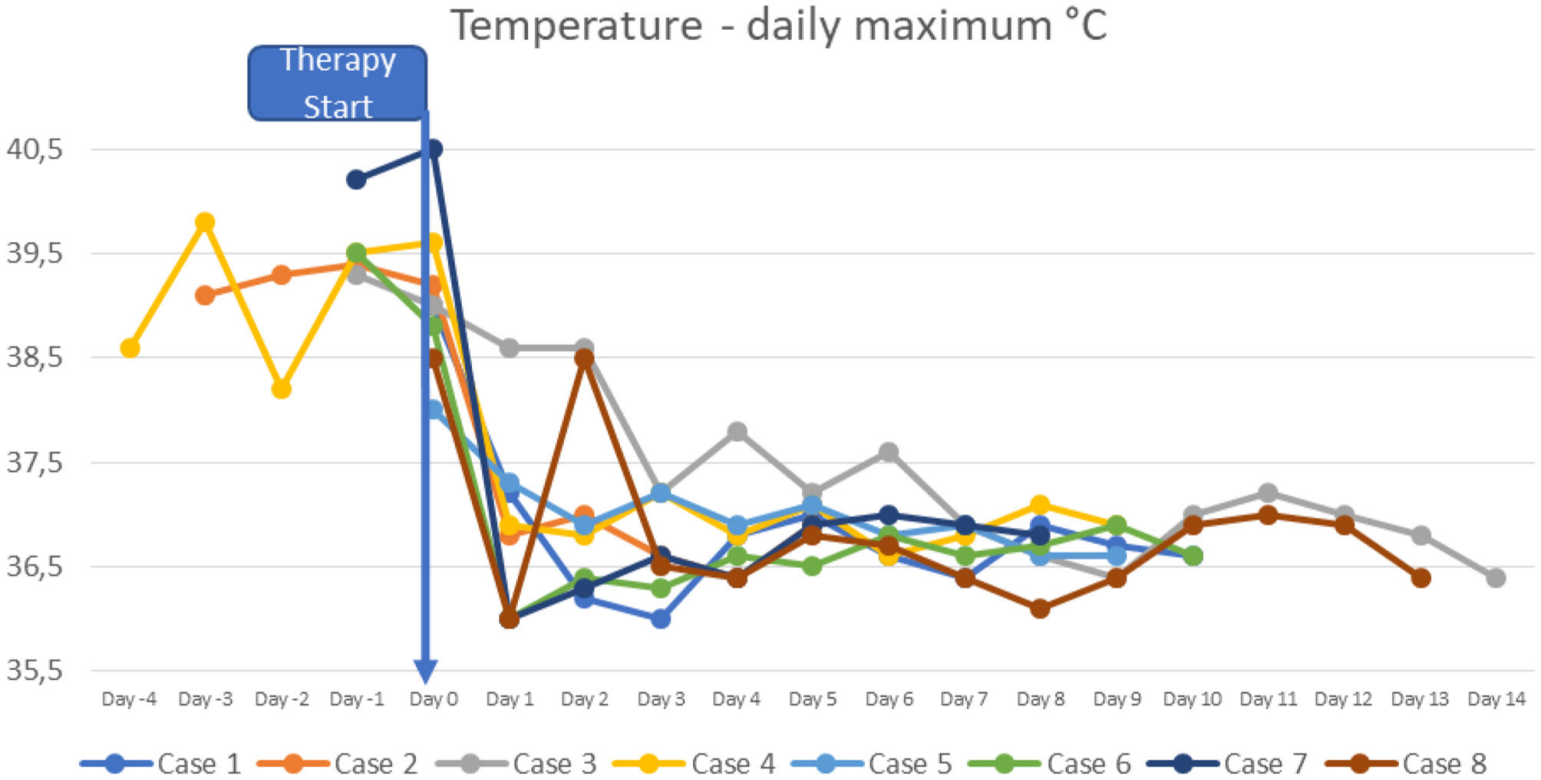
Curves of daily maximal temperature.

**Figure 2:**
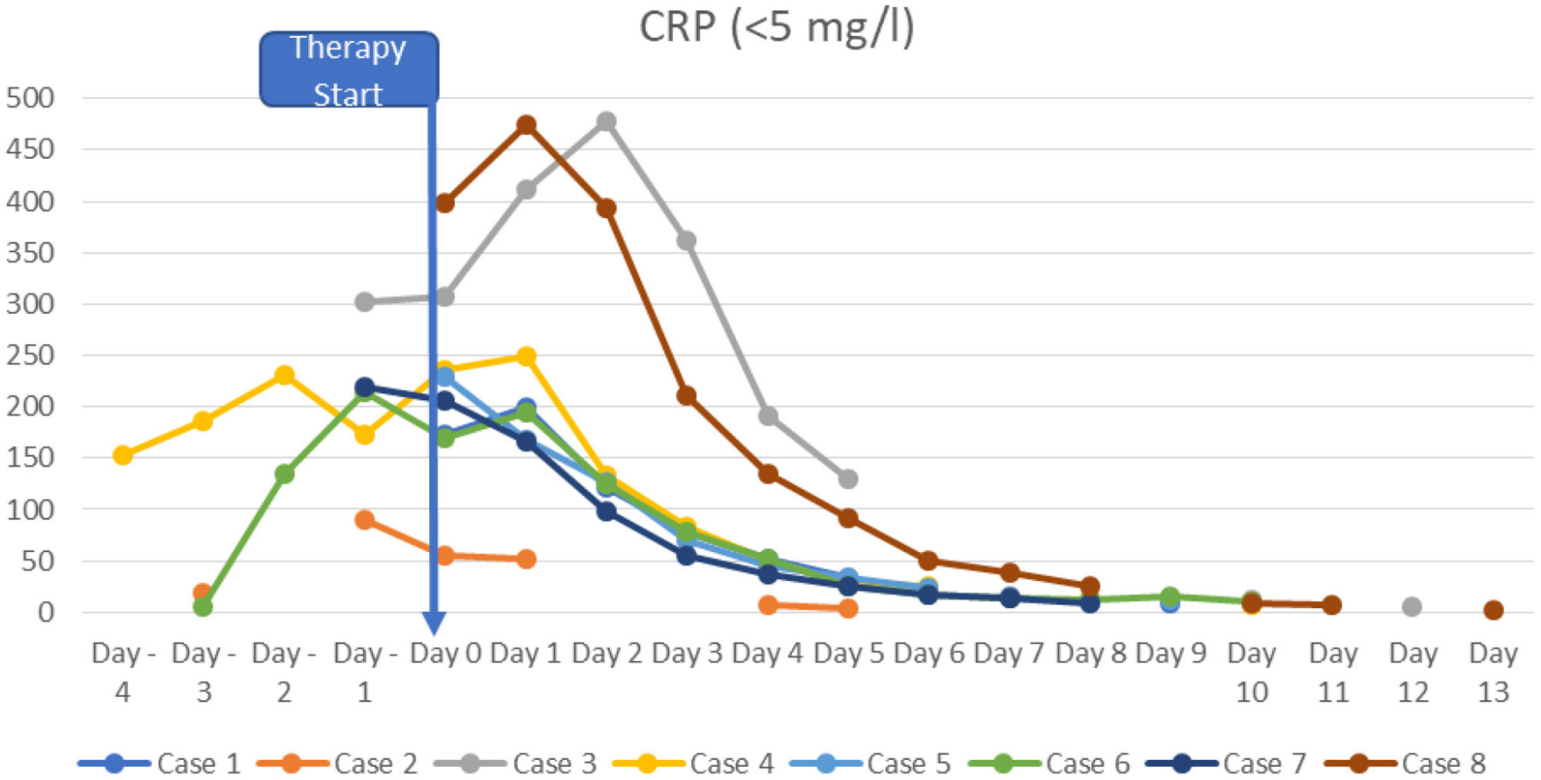
CRP.

**Figure 3:**
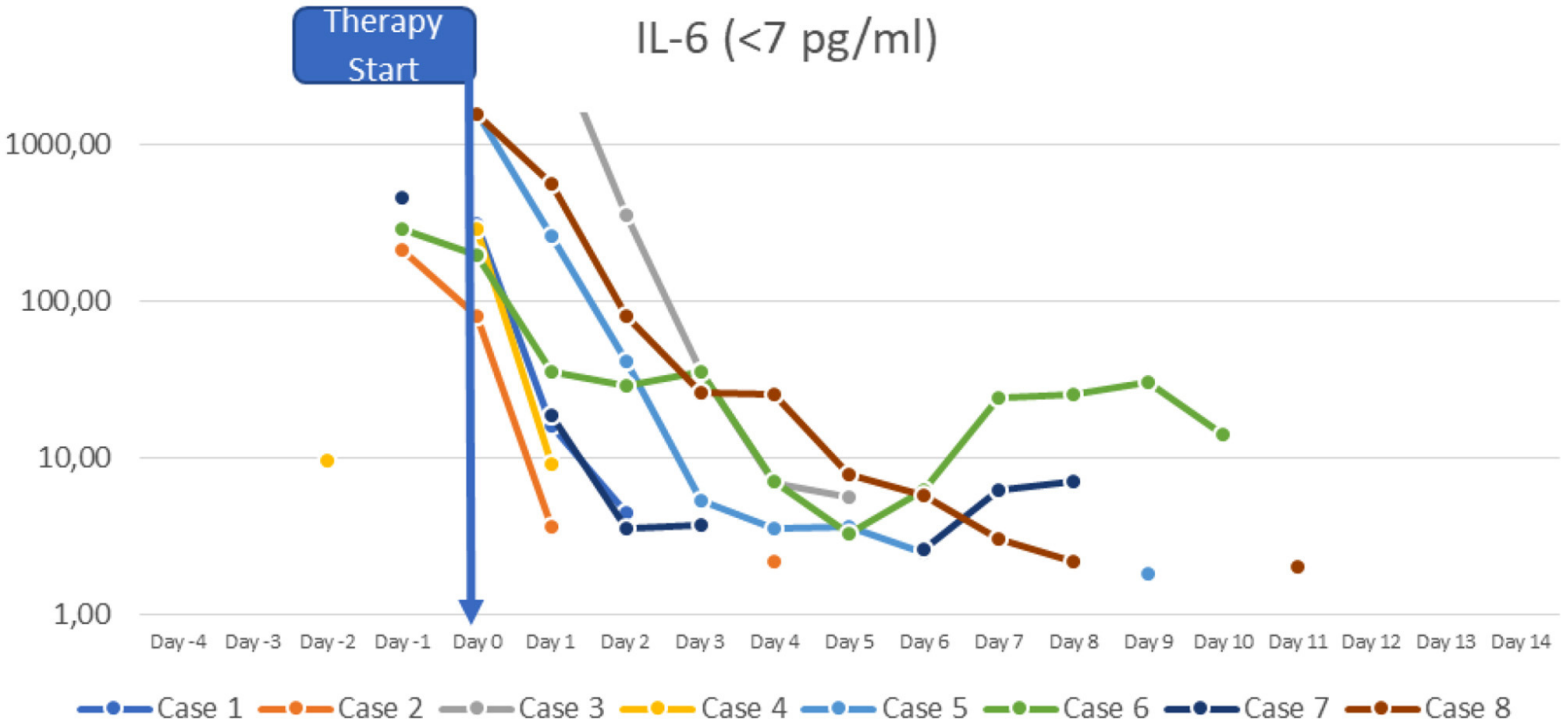
IL-6.

**Figure 4:**
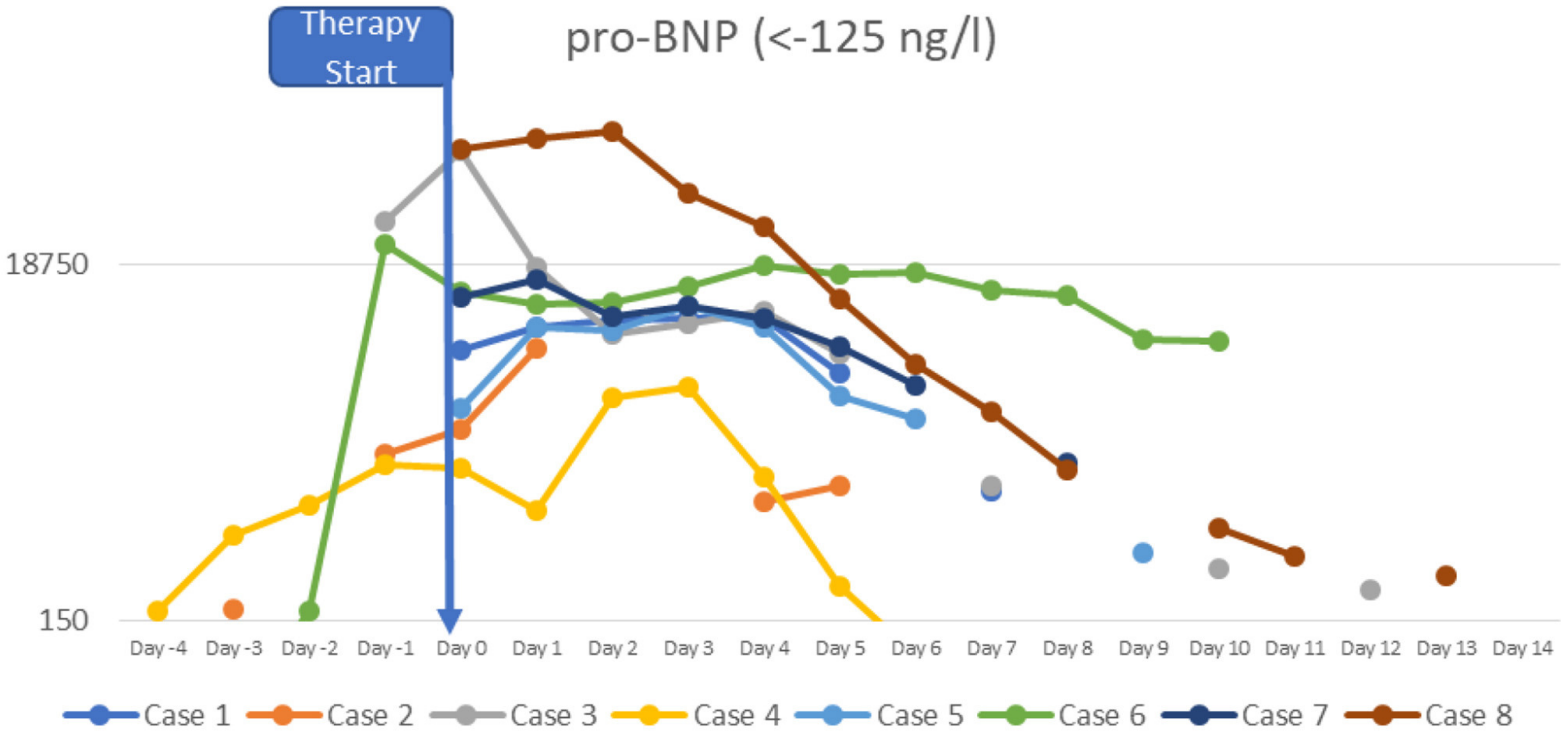
Pro-BNP.

**Figure 5:**
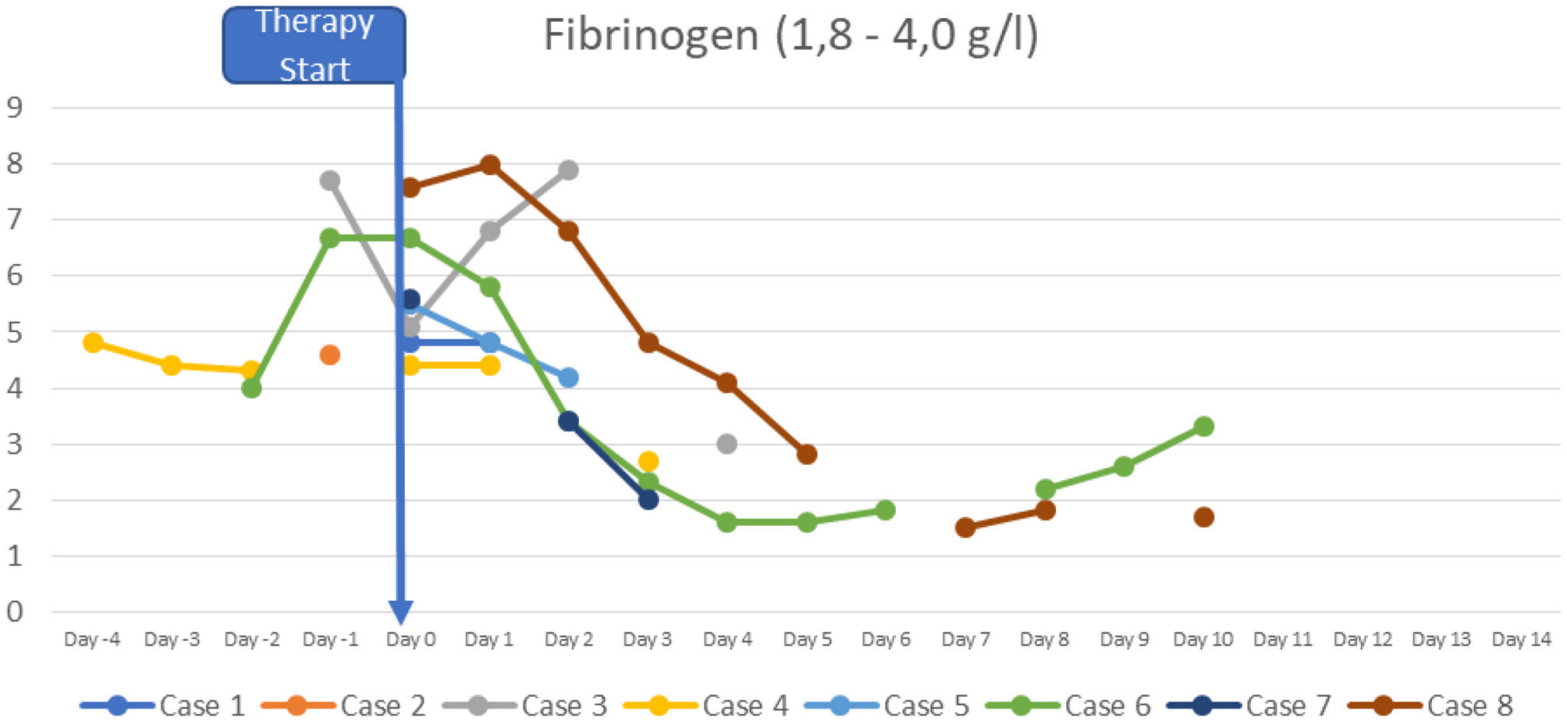
Fibrinogen.

At the time of diagnosis, the majority of the patients had normal or slightly increased leukocyte counts, with relative lymphocytopenia and relative neutrophilia, mild hyponatremia, thrombocytopenia, elevated inflammatory markers (CRP to 478.8 mg/l; IL-6 to >5,000 pg/ml; ferritin to 1,250 μg/l, sedimentation rate to >119 mm in the 1st hour, triglycerides to 380 mg/dl), hypoalbuminemia, elevated cardiac enzymes (maximum pro-BNP of 89367 ng/l, maximum high-sensitivity troponin I value of 20,308 ng/l) and elevated D-dimer. SARS-CoV-2 antibodies (IgG) were elevated in all patients (3–9 times above the cut-off; from 42 to 121 U/ml, reference value <12 negative, >15 positive). Viral or bacterial infections were ruled out in all patients. Additional details on laboratory findings, and the course of fever are presented in Figures 1–5. Echocardiography showed significantly impaired left-ventricular (LV) function in two patients [systolic function (SF) 20–25%], and moderately impaired LV function in three patients (SF 33–35%). Two patients had echogenic coronary swelling without evidence of coronary aneurysm, six patients had minor tricuspid valve regurgitation, and four had additional minor mitral valve regurgitation. Ultrasound investigation of the abdomen revealed mesenteric lymphadenitis in three patients, edema of the gallbladder wall in two patients, hepatosplenomegaly in one patient, hepatomegaly with signs of hyperemia in both testicles and epididymis in one patient. One patient underwent surgery due to suspected appendicitis, with intraoperative findings of mesenteric lymphadenitis, an ulcerous appendix and peritonitis. However, the patient's condition deteriorated after 3 days: he had fever, signs of inflammation, and elevated cardiac enzymes. The patient developed Kawasaki-like symptoms including rash, bilateral conjunctivitis, palmar edema, and injected and fissured lips.

All patients were treated with intravenous immunoglobulins (usually for 2–3 days), high doses of cortisone, high doses of aspirin (one patient was only given low doses due to severe thrombocytopenia), antithrombotic therapy with low-molecular heparin, and antibiotics. The treatment resulted in rapid clinical improvement. The patients' fever returned to normal, while inflammatory signs and elevated heart enzymes were reduced. Cardiac symptoms resolved and blood pressure returned to normal in all patients. Three patients had minor pericardial and pleural effusions at the time of discharge from the hospital. All eight patients recovered and were discharged after 9–54 days of hospitalization (median 13 days). All children are being monitored for abnormalities on echocardiography (2 weeks after discharge and once a month thereafter).

## Diagnosis

As this syndrome is new and a notable number of patients were admitted to our hospital within a short period of time, we developed guidelines based on the existing recommendations. In a multidisciplinary approach, we appointed a panel of expert pediatricians (pediatric cardiologists, pediatric rheumatologists, and pediatric intensive care physicians).

Based on the new guidelines, patients with suspected MIS-C undergo extensive diagnostic tests to corroborate the suspected diagnosis and rule out other causes such as bacterial infection (Table 3).

**Table 3:** Diagnostic approach.

Extended blood tests	Electrolytes (signs of hyponatremia), glucose, vitamin D, SarsCoV-2 IgG
	Full blood count (signs of Lymphopenia, neutrophilia, thrombocytopenia)
	Inflammatory markers: procalcitonin, IL-6, ferritin, CRP, erythrocyte sedimentation rate
	Cardiac enzymes: Pro-BNP, trop-I, CK-MB, CK
	creatinine, BUN, uric acid, GOT, GPT, GGT, AP, cholinesterase, lipase, bilirubin, albumin, total protein, LDH
	Coagulation tests (including fibrinogen, D-dimer)
Microbiological testing	Blood culture, urine culture, stool culture, PCR for respiratory viruses and cultures of a throat swab
Mandatory diagnostic procedures	12-channel ECG and echocardiography
Conditional diagnostic procedures	Chest X-ray, abdominal ultrasound
Vital signs monitoring	ECG, O2 saturation, and non-invasive blood pressure control.

## Treatment

Simultaneous administration of the medications listed in Table 4 was started in all eight patients with MIS-C.

**Table 4:** Therapy approach.

I	Immunoglobulins	1 cycle of 2 g/kg body weight given in 2 days
II	Cortisone	Methylprednisolone 20–30 mg/kg/day (maximum 1 g/day) for 3 days, then 1–2 mg/kg/d until day 7, then linear reduction over 2–3 weeks
III	Acetylsalicylic acid	30–50 mg/kg/day until the patient has no fever for 48 h, then a low dose 3–5 mg/kg/day for 6 weeks
IV	Antibiotics	Piperacillin/tazobactam (preferred in cases of abdominal problems), cefotaxim (along with daptomycin), additional clindamycin in cases of signs of shock
Adjuvant therapy	Gastric protection	PPI 1–2 mg/day until cortisone is reduced
	Thrombosis prophylaxis	Low-molecular heparin or continuous heparin
	Biologic drugs	IL-1 inhibitor (anakinra), or IL-6 inhibitor (tocilizumab), or TNF-α inhibitor (infliximab)

A single course of immunoglobulins (2 g/kg body weight) was divided into two doses of 1 g/kg body weight, administered on 2 consecutive days. Intensive care physicians cautioned against administering the entire amount in a single dose due to high viscosity and potential volume overload.

Concomitantly, a high dose of methylprednisolone (20–30 mg/kg body weight) was administered for 3 days, reduced to 2 mg/kg body weight until day 7, and then tapered over 2–3 weeks.

Acetylsalicylic acid (ASA) was started at a high dose of 30–50 mg/kg body weight, divided into four doses until the patients had no fever for at least 48 h. The dose was then reduced to 3–5 mg/kg body weight for a minimum of 6 weeks, depending on the outcome of echocardiography. Patients with severe thrombocytopenia received low-dose aspirin from the very start.

Antibiotics were aligned to the patient's resistance. In patients with signs of abdominal involvement, we give preference to piperacillin/tazobactam at our hospital. In all other cases, we used cefotaxim (optionally additional daptomycin). Patients with signs of (toxic) shock received additional clindamycin.

A proton pump inhibitor (PPI) was given for gastric protection. Furthermore, we considered thrombosis prophylaxis when the patient had an intravenous line, was immobile, of post-pubertal age, or on estrogen therapy.

In the event of no response to treatment, we recommend working with a pediatric rheumatologist to determine a potential second course of immunoglobulins or consider treatment with biologic drugs (such as an IL-6 inhibitor, a TNF-α inhibitor, or an IL-1 inhibitor).

Children should be treated in a hospital setting equipped with intensive-care facilities because the patient's clinical condition is liable to deteriorate rapidly at any time.

Pediatric cardiologists and rheumatologists should be involved in decisions regarding the treatment and in follow-up investigations. After discharge from the hospital, patients were advised against strenuous physical activity for at least 3–4 months (in accordance with the guidelines for myocarditis or Kawasaki disease with cardiac impairment).

## Discussion

SARS-CoV-2, a novel virus, was considered nearly harmless for children. However, from April 2020 onward we have had increasing reports of a new life-threatening entity named MIS-C. After the highest SARS-CoV-2 infection rate registered in Austria in November 2020 (11), a significant number of MIS-C patients were treated at Klinik Donaustadt in Vienna in December 2020.

The treatment approaches mentioned in the existing guidelines and published recently from the UK, Germany and Switzerland differ in terms of phenotypes (10, 12–17). All patients who reported at our center fulfilled the diagnostic criteria of MIS-C in accordance with the WHO definition, as well as the PIMS-TS criteria.

In most cases, we were unable to distinguish between the “non-specific” and the “Kawasaki-disease-like” phenotype at the time of admission. The patients presented with fever, and the majority of them also had abdominal pain, diarrhea, and/or emesis. Only one patient had Kawasaki-like signs. Six of the eight patients with a “non-specific” phenotype at admission developed signs of the “Kawasaki-disease-like” phenotype during the following days.

Thrombocytopenia in MIS-C as opposed to thrombocytosis in Kawasaki disease, a normal leukocyte count with relative lymphocytopenia and neutrophilia in MIS-C as opposed to leukocytosis in Kawasaki disease, help to distinguish these entities. Elevated D-dimer levels and highly elevated pro-BNP are further laboratory characteristics of MIS-C. All eight patients had positive SARS-CoV-2 IgG antibodies as evidence of a past SARS-CoV-2 infection. Two of the eight patients had a prior history of a mild COVID-19 infection (median 3.5 weeks before signs of MIS-C), one had been asymptomatic, family members of three patients had tested positive, and two patients had no history of a COVID-19 infection.

The most common reason for admission to the pediatric ICU was significantly reduced blood pressure and the need for vasopressor or vasoactive support; one patient needed invasive ventilation.

In view of the fact that MIS-C must be identified and treated early, we started therapy regardless of the phenotype. Immunoglobulins, cortisone, and acetylsalicylic acid were given simultaneously; the treatment was successful in all cases. This treatment approach has also been confirmed recently in a retrospective study (18). None of our patients failed to respond, and none needed a second cycle of immunoglobulins or biologic drugs. In rare cases, patients were reported to be unresponsive to the first cycle of immunoglobulins (19). We registered a rapid reduction of inflammatory signs, resolution of fever and hypotension, and improvement of cardiac function. Furthermore, the treatment was well-tolerated and no significant side effects were registered. All patients were discharged from the hospital without sequelae. However, in view of the small number of our series, the favorable outcome must be viewed with caution. In contrast, by mid-January 2021, 26 children in the USA had died due to complications of MIS-C (20).

The use of high-dosed acetylsalicylic acid at the start of treatment is a debated issue. The relevance of ASA in preventing coronary aneurysm was questioned in recent studies, but ASA is still included in the treatment guidelines for Kawasaki disease (21–23). In the US study, as many as one third of children with MIS-C developed coronary artery dilatation (24). Although none of the patients in our series developed coronary aneurysm, we do not know whether this was due to the natural course of the disease, the ASA therapy, or the rapid, and simultaneous start of IVIG and cortisone.

One of the primary limitations of the present report is its retrospective nature. Furthermore, it is a single-center study confined to cases registered in the eastern part of Austria. However, the multiethnic origin of the eight patients reflects the general population of two million in the city of Vienna. Being a single-center report, all patients were diagnosed and treated in the same manner.

## Conclusion

At the present time, it would be advisable to adopt a pragmatic therapeutic approach in the treatment of MIS-C: immunoglobulins in conjunction with high-dose cortisone and ASA, along with antibiotics. In a series of eight patients in Vienna, Austria, this treatment regimen was effective and well-tolerated. All patients recovered fully. The disclosure of pathophysiological pathways might explain the organotropism of the disease. Analyzing MIS-C patients on a multicenter basis will help to identify subgroups and provide targeted therapy.

## Data Availability Statement

The original contributions presented in the study are included in the article/supplementary material, further inquiries can be directed to the corresponding author/s.

## Ethics Statement

Written informed consent was obtained from the individual(s), and minor(s)' legal guardian/next of kin, for the publication of any potentially identifiable images or data included in this article.

## Author Contributions

HK planned the case series. HK and TG wrote the manuscript. All authors contributed to the article and approved the submitted version.

## Conflict of Interest

The authors declare that the research was conducted in the absence of any commercial or financial relationships that could be construed as a potential conflict of interest.
